# The Role of Oxidative Stress and Antioxidants in Liver Diseases

**DOI:** 10.3390/ijms161125942

**Published:** 2015-11-02

**Authors:** Sha Li, Hor-Yue Tan, Ning Wang, Zhang-Jin Zhang, Lixing Lao, Chi-Woon Wong, Yibin Feng

**Affiliations:** School of Chinese Medicine, Li Ka Shing Faculty of Medicine, The University of Hong Kong, Hong Kong, China; lishasl0308@163.com (S.L.); hoeytan@connect.hku.hk (H.-Y.T.); ckwang@hku.hk(N.W.); zhangzj@hku.hk (Z.-J.Z.); lxlao1@hku.hk (L.L.); vcwwong@hku.hk (C.-W.W.)

**Keywords:** oxidative stress, antioxidant, liver diseases, foods, medicinal plants

## Abstract

A complex antioxidant system has been developed in mammals to relieve oxidative stress. However, excessive reactive species derived from oxygen and nitrogen may still lead to oxidative damage to tissue and organs. Oxidative stress has been considered as a conjoint pathological mechanism, and it contributes to initiation and progression of liver injury. A lot of risk factors, including alcohol, drugs, environmental pollutants and irradiation, may induce oxidative stress in liver, which in turn results in severe liver diseases, such as alcoholic liver disease and non-alcoholic steatohepatitis. Application of antioxidants signifies a rational curative strategy to prevent and cure liver diseases involving oxidative stress. Although conclusions drawn from clinical studies remain uncertain, animal studies have revealed the promising *in vivo* therapeutic effect of antioxidants on liver diseases. Natural antioxidants contained in edible or medicinal plants often possess strong antioxidant and free radical scavenging abilities as well as anti-inflammatory action, which are also supposed to be the basis of other bioactivities and health benefits. In this review, PubMed was extensively searched for literature research. The keywords for searching oxidative stress were free radicals, reactive oxygen, nitrogen species, anti-oxidative therapy, Chinese medicines, natural products, antioxidants and liver diseases. The literature, including ours, with studies on oxidative stress and anti-oxidative therapy in liver diseases were the focus. Various factors that cause oxidative stress in liver and effects of antioxidants in the prevention and treatment of liver diseases were summarized, questioned, and discussed.

## 1. Introduction

Free radicals are atoms or molecules that have unpaired electrons, usually unstable and highly reactive [1]. In biology system, oxygen based radicals and nitrogen based radicals are two types of free radicals. Oxygen free radicals, such as superoxide, hydroxyl radicals, and peroxyl radicals, with the addition of non-radicals, such as hydrogen peroxide, hypochlorous acid and ozone, are known as reactive oxygen species (ROS), which are generated during the metabolism process of oxygen. Reactive nitrogen species (RNS), including nitrogen based radicals and non-radicals, such as nitrogen dioxide, nitric oxide radicals and peroxynitrite, are derived from nitric oxide and superoxide via inducible nitric oxide synthase (iNOS) and nicotinamide adenine dinucleotide phosphate (NADPH) oxidase, respectively [2,3]. Due to their special chemical characteristics, ROS/RNS can initiate lipid peroxidation, cause DNA strand breaks, and indiscriminately oxidize virtually all molecules in biological membranes and tissues, resulting in injury. However, since the body is able to remove ROS/RNS to a certain degree, these reactive species are not necessarily a threat to the body under physiological conditions [3,4]. As a matter of fact, ROS are required at certain level in the body to perform its important physiological functions. The generation of ROS is a natural part of aerobic life, which is responsible for the manifestation of cellular functions including signal transduction pathways, defense against invading microorganisms and gene expression to the promotion of growth or death [1]. Oxidative/nitrosative stress represents the bodies’ imbalance in the production and the elimination of reactive oxygen and nitrogen species as well as decreased production of antioxidants. In terms of oxidative stress, in specific physiological conditions, it is actually useful. For example, it could strengthen biological defense mechanisms during appropriate physical exercise and ischemia, and induce apoptosis to prepare the birth canal for delivery [2,3]. However, this is confined to particular situations, and in most other cases, large levels of ROS and oxidative stress will induce cell death through necrotic and/or apoptotic mechanisms, leading to cellular and tissue injury.

**Figure 1:**
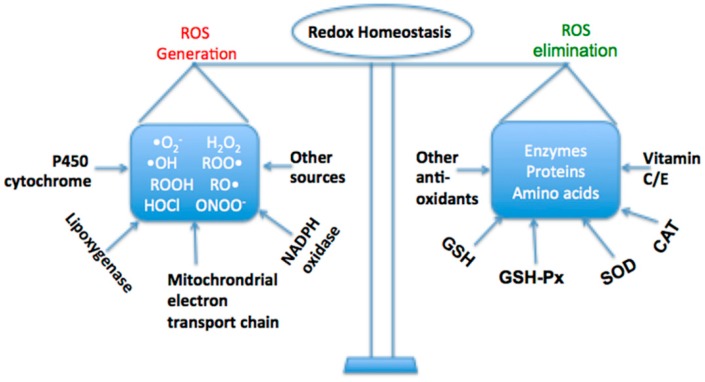
The redox homeostasis in the liver.

**Figure 2:**
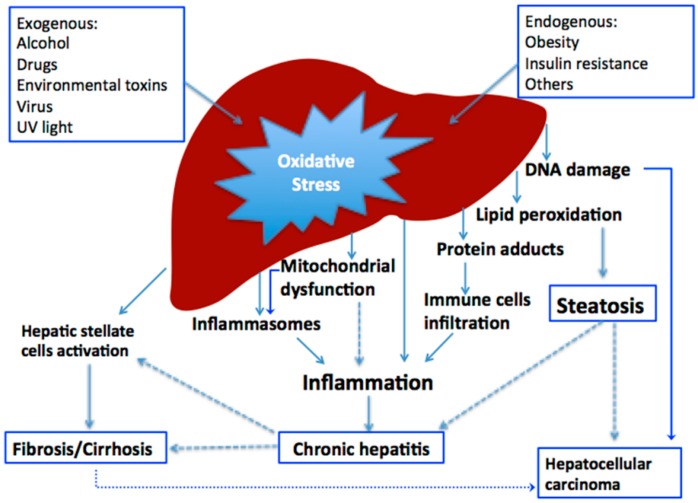
The general mechanism scheme of oxidative stress induced by various factors on liver disease.

Liver is a major organ attacked by ROS [5]. Parenchymal cells are primary cells subjected to oxidative stress induced injury in the liver. The mitochondrion, microsomes and peroxisomes in parenchymal cells can produce ROS, regulating on PPARα, which is mainly related to the liver fatty acid oxidation gene expression. Moreover, *Kupffer* cells, hepatic stellate cells and endothelial cells are potentially more exposed or sensitive to oxidative stress-related molecules. A variety of cytokines like TNF-α can be produced in *Kupffer* cells induced by oxidative stress, which might increase inflammation and apoptosis. With regard to hepatic stellate cells, the proliferation and collagen synthesis of hepatic stellate cells is triggered by lipid peroxidation caused by oxidative stress [6,7,8]. In mammals, a sophisticated antioxidant system has been developed to maintain the redox homeostasis in the liver (Figure 1). When the ROS is excessive, the homeostasis will be disturbed, resulting in oxidative stress, which plays a critical role in liver diseases and other chronic and degenerative disorders [9]. The oxidative stress not only triggers hepatic damage by inducing irretrievable alteration of lipids, proteins and DNA contents and more importantly, modulating pathways that control normal biological functions. Since these pathways regulate genes transcription, protein expression, cell apoptosis, and hepatic stellate cell activation; oxidative stress is regarded as one of the pathological mechanisms that results in initiation and progression of various liver diseases, such as chronic viral hepatitis, alcoholic liver diseases and non-alcoholic steatohepatitis [10,11]. It has also been suggested that there are complicated cross-talks among pathological factors, inflammation, free radicals and immune responses [11,12]. The general mechanism scheme of oxidative stress induced by various factors on liver disease is concluded in Figure 2. Moreover, systemic oxidative stress arising during liver disease can also cause damage to extra-hepatic organs, such as brain impairment and kidney failure [13]. It was suggested systemic oxidative stress might be a significant “first hit”, acting synergistically with ammonia to induce brain edema in chronic liver failure [14]. With regard to kidney failure, systemic oxidative stress is considered to play a critical role in the pathophysiology of several kidney diseases [15,16].

Both enzymatic and non-enzymatic antioxidant system are essential for cellular response in order to deal with oxidative stress under physiological condition. Therefore, antioxidant enzyme such as CAT, SOD, and GSH-Px and non-enzymatic electron receptors such as GSH are affected and used as indexes to evaluate the level of oxidative stress [12,17,18,19]. Notably, erythroid 2-related factor 2 (Nrf2) is a major regulator of cellular redox balance [20]. Under physiological condition, Nrf2 binds to kelch-like ECH-associated protein-1 (Keap1) in the cytoplasm, and the ones remaining are inactivated and easily to be degraded. Under oxidative stress, however, Nrf2 dissociates form Keap1 by Keap1 modification or Nrf2 phosphorylation and are thus activated. The activated Nrf2 translocates into the nucleus and interacts with antioxidant response element (ARE), promoting the expression of cytoprotective target genes including antioxidant enzymes and phase II detoxifying enzymes [21]. The enhanced activation of Nrf2 by pharmacologic molecules or genetic engineering has been shown to protect the liver in different oxidative stress models [22]. For example, in terms of pharmacologic activation of Nrf2, the use of small molecules, such as BHA, oleanolic acid, ursolic acid and CDDO-Im have been reported to show hepatoprotection against liver damage induced by acetaminophen, a famous drug possessing hepatotoxicity. During the process where mitochondria convert acetate into ATP, a significant amount of free radicals are generated, which results in cellular injuries, especially to mitochondria themselves. Activation of Nrf2 protects mitochondria from oxidative stress via a variety of mechanisms depending on different circumstances, such as increasing antioxidant levels, protecting against mitochondrial permeability transition pore opening, maintaining the mitochondrial redox state, enhancing mitochondrial biogenesis by promoting transcription of nuclear respiratory factor 1 (Nrf1). For fatty liver disease, activation of Nrf2 could facilitate fatty acid metabolism in liver by directly regulating fatty acid metabolism related genes, such as CD36 [20,22]. Furthermore, the enhanced antioxidant signaling regulated by activated Nrf2 protects mitochondria from oxidative damages, which further ensures competent hepatic fatty acid catabolism.

Regarding the vital role of oxidative stress in chain of liver diseases, various anti-oxidative therapy and antioxidants are proposed to prevent and treat liver diseases [9,12]. A series of studies have tested the effectiveness of some antioxidants in the treatment of patients with various liver diseases, such as chronic hepatitis C virus infection, alcoholic hepatitis or cirrhosis, and non-alcoholic fatty liver disease (NAFLD). The clinical effects of antioxidants as adjuvants including vitamin E/C, mitoquinone, *N*-acetylcysteine, polaprezinc silymarin, silibinin and some antioxidant cocktail on chronic hepatitis C patients have been examined has shown clear benefit of antioxidants to interferon based therapy of HCV [23,24]. However, despite some positive results were obtained, it cannot reach to the conclusion that antioxidants are useful therapeutic agents for chronic hepatitis C partly due to the sample scale and treatment duration. Vitamins E/C, *N*-acetylcysteine, polyenylphosphatidylcholine, silymarin, and antioxidants cocktail have been attempted for the treatment of alcoholic hepatitis or cirrhosis patients [24,25,26]. Although some promise has been shown, results indicated that many antioxidants failed to improve the outcome of patients [27]. Additionally, a great deal of studies has investigated the therapeutic effects of vitamins E/C and *N*-acetylcysteine on NAFLD. It is worth noting that vitamin E has been demonstrated clinically to be a rather promising drug for the treatment of non-alcoholic steatohepatitis [28,29]. Although data from clinical studies is yet to prove the efficacy of antioxidant, application of antioxidants is a rational curative strategy for prevention and treatment of liver diseases involving oxidative stress [17,30]. Natural antioxidants have been found in many edible (such as fruits, vegetables, cereals and tea) and medicinal plants, which often possess strong antioxidant and free radical scavenging abilities as well as anti-inflammatory action [9]. Several well-elaborated reviews concerning antioxidants as therapeutic agents for diverse liver diseases in clinic have been published [11,31,32], therefore, in this review, particular attention will be drawn on the factors causing oxidative stress in liver and *in vivo* effects of antioxidants for the prevention and treatment of liver diseases. Moreover, although oxidative stress has been suggested to exist in almost all liver diseases, since the fact that there are no animal models with virus-induced liver disease, including hepatitis A, hepatitis B, and hepatitis C, the role of oxidative stress in viral hepatitis are not included in this review.

## 2. Oxidative Stress in Liver Diseases

### 2.1. Oxidative Stress Caused by Alcohol

**Figure 3:**
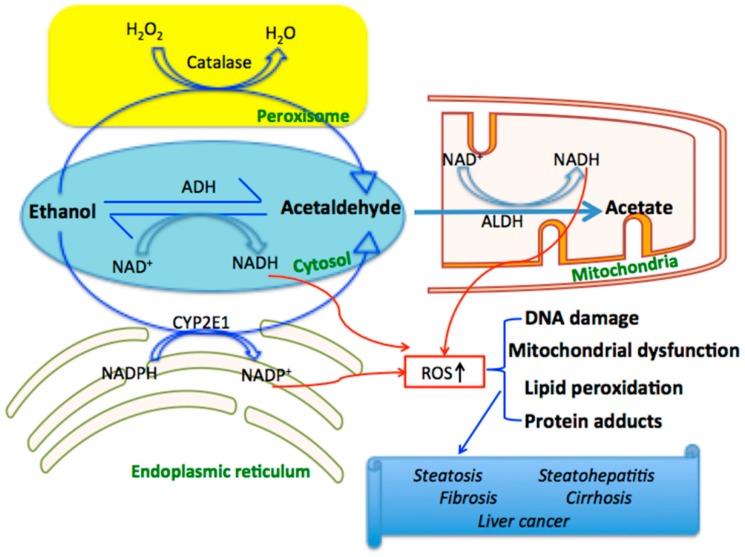
The metabolic process of ethanol in hepatocyte and the generation of ROS contributing to the liver diseases.

Alcohol beverages are widely consumed all over the world; however, excessive alcohol consumption may cause a series of health problems. It was reported that alcohol consumption accounting for an estimated 3.8% of global mortality. Alcoholic liver disease (ALD) is one of the most important causes of liver-related death, which is associated with increased dose and time of alcohol intake. In 2003, it has been reported that age- and sex-adjusted mortality rate of ALD was 4.4/100,000. Although reductions in overall ALD mortality were observed in several reports on a country scale, it is more likely due to advances in disease management rather than a decrease in the prevalence of ALD, which could be supported by increases in hospital admissions for alcoholic hepatic failure and alcoholic hepatitis [33,34,35]. ALD may progress from steatosis to more severe liver diseases form, such as hepatitis, fibrosis, and cirrhosis [36,37]. As a matter of fact, more than 90% heavy drinkers develops fatty liver, and about 30% of heavy drinkers further develops advance forms of ALD. Although pathogenesis of ALD has not been fully elaborated, the direct consequence of ethanol metabolism seems to be related to ROS production, mitochondrial injury and steatosis, which are the common features of acute and chronic alcohol exposure [32,38,39]. It is well illustrated that at least three distinct enzymatic pathways are involved in the process of ethanol oxidation [15]. The primary pathway for the ethanol metabolism is dehydrogenase system. It is initiated by alcohol dehydrogenase (ADH), a NAD^+^-requiring enzyme expressed at high levels in hepatocytes, which oxidizes ethanol to acetaldehyde. Then, acetaldehyde enters the mitochondria where it is oxidized to acetate by aldehyde dehydrogenases (ALDH). The second major pathway to oxidize ethanol is the microsomal ethanol oxidizing system (MEOS), which involves an NADPH-requiring enzyme, the cytochrome P450 enzyme CYP2E1. The MEOS pathway is prompted in individuals who consume alcohol chronically. In addition, infrequently, ethanol can also be oxidized by catalase in peroxisomes. Since this oxidation pathway requires the presence hydrogen peroxide (H_2_O_2_), under normal conditions, this pathway plays no major role in alcohol metabolism [15,16,17]. During the metabolism processes via dehydrogenase system and MEOS system, NADH or NADP^+^ will be produced in bulk, leading to the increase of ROS, which cause oxidative stress resulting in hepatocyte injury, and finally trigger various liver diseases (Figure 3).

Studies have demonstrated that enzymatic as well as non-enzymatic systems which maintaining cellular homeostasis are remarkably affected by alcohol in diverse models. In particular, the activities of SOD, CAT, GSH-Px, GRD, and GST, as well as the level of lipid peroxidation were changed in animals treated with alcohol [19,40,41,42]. For example, SOD and CAT activities were decreased and the lipid peroxidation level was significantly increased in the liver of 30 days alcohol-treated diabetic rats [40]. An increase of lipid peroxidation and hepatic cytochrome P450, and decrease of hepatic SOD, GSH-Px, GRD, GST, and GSH were also observed in mice treated with dimethoate in combination with ethanol [41]. Furthermore, oxidative stress and antioxidant enzyme were measured in patients with ALD [32]. It was found that as the severity of the disease increased, followed by elevation of serum level of lipid peroxidation indicator malondialdehyde (MDA) and the concentrations of serum vitamins E and C, which act as indexes of antioxidant status, were decreased in ALD patients. The pro-oxidant and antioxidant status in chronic alcoholics have been detected in several studies. The significant decreases of GSH levels in liver and blood of patients with alcoholic liver disease were observed when compared to controls. However, the activity/content of SOD and CAT after alcohol exposure are rather controversial, with reports of increases, no changes, or decreases, depending on the amount and time of alcohol consumption [43,44]. Nevertheless, the increased oxidative stress in patients with ALD has been demonstrated. It was argued that the increases of antioxidants enzymes such as SOD, CAT and GSH-Px might be a compensatory regulatory response to increased oxidative stress [45]. The level of ALT was increased significantly while the level of AST was decreased significantly in patients with ALD [32,46,47].

### 2.2. Oxidative Stress Caused by Drugs

The liver is the most frequently targeted organ in terms of drug toxicity. The production of radical species, specifically ROS and RNS, has been proposed as an early event of drugs hepatotoxicity and as an indicator of hepatotoxic potential [48]. It has been discovered that a lot of drugs could induce oxidative stress including increase of cellular oxidants and lipid peroxidation, depletion of antioxidants in the liver, such as anti-inflammation drugs, anti-analgesic drugs, anti-cancer drugs and antidepressants. For example, sulfasalazine, a drug to treat inflammatory bowel diseases, has been found to induce hepatic oxidative damage [49]. Oral sulfasalazine administration could reduce SOD but increase CAT activity significantly. It is also suggested that oxidative damage is involved in hepatotoxicity of sulfasalazine treatment. As for zoledronic acid, it is a nitrogen-bearing bisphosphonate, and used to treat the cancer-associated hypercalcemia. It has been shown that zoledronic acid significantly elevated MDA and nitric oxide levels, whereas reduced GSH levels, which indicated that zoledronic acid could induce oxidative stress and decrease antioxidant level in liver [18]. Furthermore, liver antioxidant capacity in hepatic injury induced by paracetamol, an extensively used analgesic compound in mice was evaluated [50]. It was shown that paracetamol induced a remarkable increase of MDA and nitrite as well as nitrate in the liver, with potent decrease of total SOD and Cu/Zn-SOD activity. Samarghandian *et al*. [51] studied effect of long-term treatment of morphine on enzymes, oxidative stress indices and antioxidant status in male rat liver. The results showed that the levels of ALT, AST and lactate dehydrogenase (LDH) in serum as well as MDA in liver were significantly elicited, while the activities of SOD, glutathione-s-transfrase and CAT were remarkably reduced by morphine. Oxidative stress generated by anticancer drugs including doxorubicin, paclitaxel and docetaxel in the liver of rats have been indicated. It was found that all three drugs increased thiobarbituric acid-reactive substances (TBARS), and the administration of docetaxel significantly decreased the activity of SOD. Furthermore, combined administration of two drugs generated greater changes in oxidative stress related molecules than single agents [52]. Nimesulide, nonsteroidal anti-inflammatory drug, could increase the activities of ALT, AST, ALP and the content of bilirubin in the serum. The activities of SOD and CAT and GSH-Px in the liver were decreased by nimesulide in mice [53]. Chronic administration of fluoxetine (15 mg/kg/day) or clozapine (20 mg/kg/day) was measured in rats exposed to chronic social isolation and controls. The increased serum ALT activity, MDA, decreased GSH levels and compromised SOD expression suggests a link between drugs and hepatic oxidative stress [54]. Anti-tuberculosis agent isoniazid (INH) resulted in both oxidative and nitrosative stress, but the correlation of hepatotoxicity severity with RNS rather than ROS suggested that ONOO^−^ generation and mitochondrial dysfunction are responsible mechanisms for hepatotoxicity of INH *in vivo* [55,56].

Although hepatotoxicity induced by various drugs in humans has been demonstrated in a great number of clinical trials, report concerning the role of oxidative stress in patients with drug induced liver disease is limited by far. For example, mitochondrial dysfunction and DNA damage are found to be critical events in the underling mechanism of paracetamol induced hepatotoxicity in patients, which is supposed to partly attribute to oxidative stress, but, accurate and direct evidence to show the status and role oxidative stress in patients is lacking [57]. As a matter of fact, currently, in addition to animal model study, the investigation of hepatotoxicity induced by drugs is mainly based on the results of retrospective study, whereas there are few clinical studies with large numbers of patients. Moreover, models using human cells have been attempted to mimic pathogenesis of drug induced hepatotoxicity in humans [55]. Overall, clinical data and appropriate experimental model, which could closely resemble the human pathophysiology, is critical for future study of antioxidant treatment for hepato-toxicity caused by drugs.

### 2.3. Oxidative Stress Caused by Environmental Pollutants

Environmental pollutants such as heavy metals and microcystin have been shown to cause oxidative damage in liver of animal models. Antioxidant defense system in rat liver was damaged after mercury chloride treatment [58]. Mercury chloride at the dose of 0.1 mg/kg could induce a significant decrease in both Mn-dependent SOD and Cu- and Zn-dependent SOD activities, and progressive changes of CAT, GSH-Px, GRD and glucose-6-phosphate dehydrogenase activities. This is also accompanied by a minor increase in serum ALT and γ glutamyltransferase. The results showed that low dose of mercury could incur oxidative stress and hepatic damage. Besides mercury, lead was also found to exacerbate liver lipid peroxidation in protein-undernutrited rats, in which the study also suggested that free radicals is a pathological mechanism for hepatotoxicity of lead [59]. Microcystins are algae toxins produced by cyanobacteria, kind of cyclic nonribosomal peptides, possessing hepatotoxicity that may cause severe injury to the liver. The effect of microcystin LR, the most studied toxic variants, on antioxidant enzymes and lipid peroxidation was investigated in liver rats after acute exposure [60]. The reduction of enzymes activities of GSH-Px, GRD, SOD and CAT as well as significant increase of lipid peroxidation levels were observed in the liver of microcystin LR-treated rat. These results showed that acute exposure of microcystin LR could result in perturbation of the antioxidant enzymes, suggesting the involvement of oxidative stress in the pathogenesis of microcystin LR-induced toxicity.

### 2.4. Oxidative Stress Caused by Other Factors

Other factors such as radiation and temperature may also induce hepatic oxidative stress. The oxidative stress induced through exposure of mobile phone-like radiation has been investigated in the liver of guinea pigs [61]. The results showed that after radiation exposure, the levels of MDA and total nitric oxide were significantly increased and the activities of SOD, myeloperoxidase and GSH-Px were reduced in the liver of guinea pigs. Additionally, the severity of oxidative damage was increased along with the duration of radiation exposure. The results suggested that mobile phone-like radiofrequency radiation could induce oxidative damage in liver, implying the adverse effect of mobile phone use. Moreover, study observed that cold stress could lead to decrease in CAT, SOD and GSH-Px activities in rat liver when the rats were kept at 10 °C for a week, which indicated that cold stress may cause hepatic damage which is associated with oxidative stress [62].

Benzoyl peroxide is a substance with strong oxidizing capacity, and broadly used as flour bleaching agent. The hepatic antioxidant status and ATPases were affected by benzoyl peroxide in mice [63]. Following benzoyl peroxide exposure, SOD activity was reduced significantly, whereas the content of MDA was increased in liver tissue. The activities of Ca^2+^-ATPase and Mg^2+^-ATPase in liver were also significantly decreased by benzoyl peroxide. In another study, the effect of ZnO_2_ nanoparticles, a common cosmetic component, on cellular oxidative stress in mouse liver was investigated [64]. After exposure to ZnO_2_ nanoparticles, viability of hepatic cells was decreased in concentration-dependent manner, and decrease in antioxidant enzyme levels as well as increase in DNA adduct.

Studies have suggested that maternal high-fat diet feeding could raise the incidence of metabolism-related diseases in offspring, including chronic liver disease. Zhang *et al*. [65] found that maternal high-fat diet increased the level of plasma triglyceride and hepatic TBARS significantly. The size of lipid droplets in the liver of rat offspring was also increased. Expression of antioxidant defense genes, such as GSH-Px-1, Cu/Zn-SOD, and paraoxonase enzymes, were significantly lowered in the liver. Up-regulation of the inhibitor of cyclooxygenase-2 and cyclin dependent kinase 4a, and down-regulation of cyclin D1 and phosphorylation of retinoblastoma protein were found in the offspring. These results suggested that maternal high-fat diet might reduce the capacity of antioxidant defense and speed up cellular senescence in hepatic tissue of older offspring. In another study, the effect of high dietary salt on hepatic antioxidant defensing enzyme of fructose-fed rats was investigated [66]. Feeding fructose-fed rats with high-salt diet could trigger hyperinsulinemia and insulin resistance resulting in membrane perturbation. This potentially enhanced hepatic lipid peroxidation in the presence of steatosis, and led to decrease in antioxidant defenses, as observed by reduction of GSH, SOD and CAT activities. These results indicated that consumption of salt-rich diet by insulin-resistant subjects could lead to sodium reabsorption, which may aggravate hepatic lipid peroxidation related to damage antioxidant defenses.

In addition to those liver injury induced by exogenous substances, hepatic oxidative stress has been revealed in other liver diseases and functional disorders. For instance, Messarah *et al.* [67] has found that thyroid dysfunction would increase lipid peroxidation and oxidative stress status in rat liver. In another study, oxidative stress and antioxidant status in patients with autoimmune cholestatic liver diseases (AC) or autoimmune hepatitis (AIH) were investigated [68]. Several markers of oxidative injury and antioxidant components in whole blood, serum, and urine of 49 patients with AC and 36 patients with AIH as well as healthy subjects were assessed. The results showed that both AC and AIH patients had increased levels in oxidation products of lipid and protein while significant decreased of whole blood GSH level. Protein carbonyl and isoprostane levels were increased and GSH level was gradually decreased with disease severity level (mild to severe fibrosis and cirrhosis) in both AC and AIH patients. In addition, AIH patients had higher levels of aldehydes and GSH-Px activity and lower protein carbonyl levels compared to AC patients. In patients with nonalcoholic fatty liver disease (NAFLD), the oxidative stress and antioxidant status were changed as well [69]. It was shown that level of TBARS in NAFLD patients was significantly higher than subjects with viral hepatitis or healthy controls. Moreover, the ferric reducing ability of plasma in patients with NAFLD was significantly higher than healthy controls, and diseased control group of patients. These results implied that lipid peroxidation and oxidative stress were significantly increased in patients with NAFLD. Although existence of hepatic oxidative stress in various liver diseases was commonly observed, the relationship between oxidative damage and diseases are causal and not strictly defined.

## 3. Antioxidants for Prevention and Treatment of Liver Diseases

### 3.1. Antioxidants for Prevention and Treatment of Alcoholic Liver Diseases

An obvious avenue of alcoholic liver diseases (ALD) prevention would be abstinence; however, abstinence is not easy to maintain due to the high rate of recidivism in alcoholics [14]. As mentioned above, ALD develops from simple steatosis to more severe disease forms including hepatitis, fibrosis, cirrhosis, and even hepatocellular carcinoma, which implies that preventing disease development at the early stage would be more effective than receiving treatment at end-stage of liver disease. Notably, TNF, a group of cytotoxic pro-inflammatory cytokines, is thought to play a vital role in initiation of liver damage [70]. Increasing evidence has indicated that oxidative stress might act together with endotoxins to increase TNF production. Increased circulating TNF-α stimulates TNF-α receptors of cell surface, which leads to activation of the stress-related protein kinases JNK and IKKβ, resulting in increased production of additional inflammatory cytokines, and reduced insulin sensitivity. Consequently, the inhibition of TNF is regarded as a therapy to block fatty liver and relieve liver injury [70,71]. Pharmacological and genetic manipulation of TNF have been attempted to treat liver disease. For example, anti-TNF antibodies or knocking out TNF-R1 have been treated to mice to protect against the development of ALD. However, since liver regeneration requires low “basal” contents of TNF, down regulating but not blocking totally TNF activity is a preferred therapeutic intervention for liver disease [71,72]. With better understanding of the mechanism that regulates the initiation and advancement of ALD, antioxidant therapy could be developed as directed therapy to prevent or treat ALD [32,37,73,74]. It has been demonstrated that many food and plants, such as vegetables, fruits, tea, cereals, medicinal plants, microalgae, edible macro-fungi, and wild flowers, have abundant natural antioxidants, and possess the ability of eliminating free radicals and protecting the liver from oxidative stress [75,76,77,78,79,80,81,82,83], and thus might be beneficial for liver diseases.

In recent years, a great number of natural plants has been attempted to eliminate hepatic damage induced by ethanol in animal models, and the bioactive compounds that are responsible for relieving oxidative stress are usually indistinctly ascribed to polyphenols and flavonoids compounds [42,84,85,86,87]. For example, it has been found that green tea, containing abundant water-soluble antioxidants, showed positive effect on the antioxidant abilities in rat liver with chronic ethanol treatment [84]. It was shown that significant reduction of enzymatic and non-enzymatic antioxidants levels, as well as increased levels of lipid and protein modifications was induced by ethanol diet. After administration of green tea, interestingly, the enzymes activity and level of non-enzymatic antioxidants as well as lipid and protein oxidation products were partly normalized. The effects of some natural products on hepatic alcoholic damage associated with oxidative stress were summarized in Table 1, which indicate that anti-oxidative treatment is an encouraging method to reduce alcoholic liver injury. Besides phenolic compounds, more specific bioactive compounds should be further identified and isolated in the future.

**Table 1:** The effects of antioxidants/plants on alcoholic liver damage. Up-arrow means increase and up-regulation, and down-arrows means decrease and down-regulation.

Models (Prevent/Treatemnt)	Materials	Effect	Dose (Dose-Effect)	Bioactive Compounds	References
Rats treated with ethanol diet (Prevent)	Green tea	↑ Enzymes, non-enzymatic antioxidants; ↓ lipid and protein oxidation	7 g/L in ethanol Lieber-DeCarli diet	Epicatechin, epicatechin gallate	[84]
Rats treated with ethanol (Prevent)	*Ziziphus mauritiana* leaf	↓ ALT, AST, ALP, total bilirubin, CAT; ↑ GSH-Px, glutathione reductase and SOD	200 and 400 mg/kg b.w. (Dose-effect)	Tannins, saponins and phenolic compounds	[42]
Rats sub-chronically exposed to ethanol (Prevent)	*Amaranthus hypochondriacus* seed	↓ MDA, NADPH; ↑ Cu, Zn-SOD	140 g/kg in diet	Total phenols	[87]
Mice with acute alcohol-induced liver injury (Prevent)	Peduncles of Hoveniadulcis	↓ ALT, AST, MDA; ↑ SOD, GSH-Px	100, 350 and 600 mg/kg b.w. (Dose-effect)	Non-starch polysaccharide	[86]
Rats treated with ehanol (Prevent)	Methanolic extract from *Hammada scoparia* leaves	↓ Aminotransferase, glycogen synthase kinase-3 β, lipid peroxidation; ↑ GSH-Px	200 mg/kg b.w.	Phenolic compounds	[85]
Mice with chronic alcoholic liver damage (Prevent)	Jujube honey	↓ Lipoprotein oxidation, AST, ALT, MAD, 8-hydroxy-2-deoxyguanosine; ↑ GSH-Px	27 and 54 g /kg b.w. (Dose-effect)	Phenolic acids	[88]
Mice with alcohol-induced hepatotoxicity (Treatment)	Freeze-dried, germinated and fermented mung bean	↑ Antioxidant levels, NO	200 and 1000 mg/kg b.w.		[89]
Chronic ethanol exposure in rats (Prevent)	Virgin olive oil	↓ Transaminases levels, hepatic lipid peroxidation; ↑ GSH-Px, SOD and CAT	5% (wt/wt) in diet	Tocopherols, chlorophyll, total polyphenols	[90]

In addition to these natural products, many single compounds have been investigated for their role in eliminating oxidative stress, such as l-theanine, vitamin E, *N*-acetyl cysteine, raxofelast and betaine [91]. l-theanine, a unique amino acid in green tea, has been proven to possess the ability to prevent alcoholic hepatic damage via augmenting antioxidant capacities [91]. The ethanol-stimulated increase of ALT, AST, and MDA and reduction of antioxidant enzymes activities including the activities of SOD, and CAT, as well as level of GSH were significantly inhibited by l-theanine. The regulation of l-theanine on alcohol-induced fat droplets was further confirmed by histopathological examination. Besides, vitamin E is considered to be beneficial for prevention of diseases associated with oxidative stress because of its remarkable anti-oxidative properties. Kaur *et al*. [92] has proven that vitamin E could restore the redox status, prevent oxidative stress and reduce apoptosis, and could be used as a prospective curative agent for ethanol-induced hepatic oxidative injury. Moreover, raxofelast, an analog of vitamin E, possesses the ability to inhibit lipid peroxidation in mice exposed to ethanol [93]. Raxofelast diminished the increased hepatic NF-κB activity, reduced serum ALT and liver triglycerides, lowered hepatic MAD levels, prevented liver GSH depletion, decreased Toll-like receptor-4, TNF-α, IL-6 and intercellular adhesion molecule-1 hepatic gene expression. It has been suggested that raxofelast blunted the inflammatory cascade and liver damage during chronic ethanol exposure. *N*-acetyl cysteine, a scavenger of ROS, may reverse alcoholic liver damage, and alter activities of matrix metalloproteinases [94]. Furthermore, it was shown that the ethanol-induced oxidative stress could be inhibited effectively by betaine, which is also responsible to its hepatoprotection [95].

Betulinic acid is a pentacycliclupane-type triterpene, and has a wide range of bioactivities. Yi *et al*. [96] has reported that pre-treatment of betulinic acid could significantly reduce the serum levels of ALT, AST, total cholesterol, and triacylglycerides in the mice treated with alcohol. Hepatic levels of GSH, SOD, GSH-Px, and CAT were remarkably increased, while MDA contents and microvesicular steatosis in the liver were decreased by betulinic acid. It was suggested that the hepatoprotective effect of betulinic acid is associated with the improvement of antioxidant enzymes capacity, primarily via enhancement of the tissue redox system and protection of the antioxidant system in the liver. Demethyleneberberine, a natural mitochondria-targeted antioxidant found in Chinese herb Cortex *Phellodendri chinensis*, has been demonstrated the ability of inhibiting oxidative stress and steatosis in acutely/chronically ethanol-fed mice [97].

### 3.2. Antioxidants for Prevention and Treatment of Non-Alcoholic Fatty Liver Diseases

NAFLD is characterized by abnormal fatty acids deposition in the liver cells of patients without excessive alcohol intake, viral infection or other hepatoxins, including a broad spectrum of histological irregularities [98]. Notably, obesity is considered to be the main risk factor for the development of NAFLD and the main driver of rapid rise of NAFLD prevalence [99]. The oxidative stress of endoplasmic reticulum induced by free fatty acid in the liver might contribute to the hepatic injury, progressive fibrosis and even cirrhosis [100]. In Table 2, certain antioxidants or plants were attempted to reduce liver injury induced by high fat diet in experimental animals, which indicated that most of them showed both antioxidant and hepato-protective effects. Furthermore, in a clinical trial that aims to systematically evaluate the effect of antioxidant supplements, it was found that AST levels, but not of ALT levels were reduced significantly in patients with NAFLD by antioxidant intervention. It should be pointed out that, however, data obtained is so far insufficient to figure out whether dietary supplements is beneficial or useless for patients with NAFLD [98]. To address this issue, large-scaled of prospective randomized clinical studies on this topic is quite necessary.

It has also been indicated that insulin resistance, oxidative stress, and the inflammatory cascade play a vital role in the pathogenesis of NAFLD by animal study. Data from clinic trial indicated that insulin resistance is a high risk factor of NAFLD. Recent studies have shown that insulin resistance is present in surrounding tissue and live of almost all NAFLD patients [44]. The severity of insulin resistance is correlated with the progression of disease. However, the role of oxidative stress and inflammation in the pathogenesis of NAFLD cascade need to be further studied in human. In the setting of obesity, increased fatty acids and other related metabolites enhance oxidative phosphorylation and ATP generation, leads to increase ROS/RNS production and oxidative stress. Multiple stress-sensitive kinase signaling cascades, such as JNK and IKKβ, are activated by the increased oxidative stress. Once activated, these kinases are able to phosphorylate multiple targets, including the insulin receptor and the family of IRS proteins [101]. Insulin action is impaired by the abnormal serine/threonine phosphorylation in insulin receptor and IRS proteins such as IRS-1 and IRS-2, resulting in insulin resistance. In hyperglycemia caused by insulin resistance, intensive redox reactions occur during the process of protein glycation, generating a great deal of ROS [102]. Additionally, hyperglycemia and high insulin levels stimulate fatty acids synthesis and result in increasing lipid droplets storage within hepatocytes. The excessive intracellular levels of lipid can induce hepatocytes dysfunction or death. The increased ROS also act on large molecules such as poly-unsaturated fatty acids to initiate lipid per-oxidation, which further change the fluidity and permeability of the cell membrane. The inflammatory infiltration induced by lipid per-oxidation may also result in liver inflammation and necrosis, and even fibrosis. In mitochondrion, lipid peroxidation reduces the activity of mitochondrial respiratory chain, and thereby produces more ROS and increase oxidative stress. The prolonged oxidative stress may favor insulin resistance circularly, acting like a vicious circle. Then, the persistent exposure of oxidative stress and hyperglycemia contribute to NAFLD [103,104]*.* In addition to obesity, other risk factors such as drugs, re-feeding syndrome and other disorders are considered. For example, streptozotocin-induced diabetic rats constitutes as the model of oxidative stress. It was indicated that supplementation of alpha-tocopherol increased alpha-tocopherol in liver, but not in plasma [105]. Diet supplementation of acai, a promising source of natural antioxidants, could increase mRNA levels of gamma-glutamylcysteinesynthetase and GSH-Px in liver tissue, and decrease ROS produced by neutrophils. In addition, supplementation with acai could decrease thiobarbituric acid-reactive substances levels, and increase reduced GSH content in the liver. Moreover, the effect of dietary supplementation of vitamins C and E on oxidative stress and antioxidant redox systems was studied in streptozotocin-induced aged diabetic rats [106]. GSH-Px activity and the concentration of vitamin E in liver were lower, whereas lipid peroxidation levels in liver, and contents of ALT and AST in plasma were higher in the diabetic group than in the control group and were mostly restored by vitamins C and E treatment. Furthermore, the combined treatment with vitamin C, vitamin E, and Se showed a curative effect against the liver injury in streptozotocin-induced diabetic rats [107]. The effects of some antioxidants/plants on liver of streptozotocin-induced diabetic rats are summarized in Table 3.

**Table 2:** The effects of some antioxidants/plants on NAFLD.

Models (Prevent/Treatment)	Antioxidant/Plants	Effects	Dose (Dose-Effect)	Bioactive Compounds	References
Diabetic rats fed on a high fat thermolyzed diet (Prevent)	Omega 3-polyunsaturated fatty acids	↑ SOD, CAT; ↓ triglycerides, non-esterified fatty acid, lipoperoxidation	3.0% in diet	Omega 3-polyunsaturated fatty acids	[108]
Mice fed with high-fat diet (Prevent and treatment)	*Moringa oleifera* leaves; haw pectic oligosaccharide; *Thymbra spicata*	↑ GSH; ↓ ALT, AST, ALP, lipid peroxidation	50, 150 and 300 mg/kg b.w. (No dose–effect)	Haw pectic oligosaccharide	[109,110,111]
Liver damage in diet-induced atherosclerotic rats (Prevent)	*Tulbaghia violacea* rhizomes	↓ LDH, AST, ALT, ALP, bilirubin antioxidation	100 mg/kg b.w.		[112]
Rabbits with high-fat diet (Prevent)	Apolipoprotein A–I	↑ SOD, GSH-Px; ↓ iNOS, MDA	15 mg/kg b.w.		[113]
WeRats fed a high-fat diet (Prevent)	Black cabbage sprout	↑ SOD, CAT, NADPH, GSH-Px, GRD GST	250 and 500 mg/kg b.w. (Dose–effect)		[114]

**Table 3:** The effects of some antioxidants/plants on liver of streptozotocin-induced diabetic rat.

Models (Prevent/Treatment)	Materials	Effects	Dose (Dose-Effect)	References
Streptozotocin-induced diabetic aged rats (Prevent)	Vitamins C and E	Antioxidation, hepatoprotection		[106]
Streptozotocin-induced diabetic rats (Prevent)	Acai	Antioxidation, hepatoprotection	2% (*w*/*w*) in standard diet	[115]
Streptozotocin-induced diabetic rats (Prevent)	*Herba bidentis*	Antioxidation, hepatoprotection	5 mL/kg	[116]
Streptozotocin-induced diabetic rats (Prevent)	(−)-Epicatechin	Antioxidation	15 and 30 mg/kg (Dose–effect)	[117]
Streptozotocin-induced diabetic rats (Treatment)	Stobadine		24.7 mg/kg	[118]
Streptozotocin-induced diabetic mice (Prevent)	*Terminalia glaucescens* leaves	Antioxidation	100 and 300 mg/kg (Dose–effect)	[119]
Streptozotocin-induced diabetic rats (Treatment)	Berberine	Antioxidation	75, 150 and 300 mg/kg (Dose–effect)	[120]
Streptozotocin-induced diabetic rats (Prevent)	*Aloe vera* leaves		300 mg/kg	[121]
Streptozotocin-induced diabetic rats (Treatment)	*N*-Acetylcysteine	Antioxidation	1.5 g/kg	[122]
Streptozotocin-induced diabetic rats (Treatment)	*Oroxylum indicum* stem bark	Antioxidation	250 mg/kg	[123]
Streptozotocin-induced diabetic rats (Treatment)	Maslinic acid	Antioxidation	40, 80 and 160 mg/kg (Dose–effect)	[124]
Streptozotocin-induced diabetic rats (Treatment)	Resveratrol	Antioxidation	20 mg/kg	[125]
Streptozotocin-nicotinamide induced diabetic rats (Prevent)	*Stevia rebaudiana*	Antioxidation		[126]

### 3.3. Antioxidants for Prevention and Treatment of Liver Diseases Induced by Other Factors

Since liver is an essential organ for detoxification and metabolism, and all pharmaceuticals make their way to the liver, for storage and therefore it is more prone to damage [127,128]. Paracetamol is widely used to relieve pain and reduce fever. Although use of paracetamol at its recommended dose is generally safe, overdose could still cause severe hepatic damage in many cases. As mentioned above, paracetamol may induce a remarkable increase of MDA and nitrite as well as nitrate in the liver, apart from a significant reduction in total SOD and Cu/Zn-SOD activity. Models of paracetamol-induced liver damage in mice/rats are widely used to study antioxidant and hepatoprotective effects of antioxidants/plants. For example, Rasool *et al*. [129] studied hepatoprotective and antioxidant effects of Gallic acid in paracetamol-induced liver damage in mice. It was shown that Gallic acid possessed antioxidant and hepatoprotective effects. In addition to paracetamol, some other drugs such as doxorubicin, tert-butyl hydroperoxide and d-galactosamine may also induce liver injury, which is possibly associated with the rise of oxidative stress. The effects of certain antioxidants/plants on paracetamol and other drugs-induced liver damage are summarized in Table 4. As seen from Table 4, a conclusion could be drawn that materials possessing antioxidant activity also hold capacity of hepatoprotection in animal model, which implies the correlation between antioxidative property of these compounds and their hepatoprotective effect.

**Table 4:** The effects of some antioxidants/plants on drugs-induced liver damage.

Models (Prevent/Treatment)	Materials	Effects	Dose (Dose-Effect)	References
Paracetamol-induced liver toxicity in mice (Prevent)	Gallic acid	Antioxidation, hepatoprotection	100 mg/kg	[129]
Paracetamol-induced liver toxicity in mice (Prevent)	Sauchinone	Antioxidation, hepatoprotection	30 mg/kg	[130]
Paracetamol-induced liver toxicity in mice (Prevent)	Genistein	Antioxidation, hepatoprotection	50, 100 and 200 mg/kg (Dose-effect)	[131]
Paracetamol-induced liver toxicity in mice (Prevent)	*Phyllanthus niruri*	Antioxidation, hepatoprotection	100 mg/kg	[132]
Paracetamol-induced liver toxicity in mice (Prevent)	*Polyalthia longifolia* leaves	Antioxidation, hepatoprotection	200 mg/kg	[133]
Paracetamol-induced liver damage in rats (Prevent)	*Boerhaavia diffusa* leaves	Antioxidation, hepatoprotection	100, 200, 300 and 400 mg/kg/day (No dose-effect)	[134]
Paracetamol-induced liver damage in rats (Prevent)	Saponarin from *Gypsophila trichotoma*	Antioxidation, hepatoprotection	80 mg/kg/week	[135]
Lipopolysaccharide-induced liver injury in rats (Prevent)	Carnosic acid	Antioxidation, hepatoprotection	15, 30 and 60 mg/kg (Dose-effect)	[136]
d-Galactosamine-induced liver injury in rats (Prevent)	Combination of selenium, ascorbic acid, β-carotene, and α-tocopherol	Antioxidation, hepatoprotection		[137]
d-Galactosamine-induced liver injury in rats (Prevent)	*Leucasaspera*	Antioxidation, hepatoprotection	200 and 400 mg/kg (No dose-effect)	[138]
d-Galactosamine-induced liver injury in rats (Prevent)	Swertiamarin from *Enicostemma axillare*	Antioxidation, hepatoprotection	100 and 200 mg/kg (No dose-effect)	[139]
Lipopolysaccharide/d-galactosamineinduced liver injury in rats (Prevent)	Curcumin	Antioxidation, hepatoprotection	100 mg/kg	[140]
Lipopolysaccharide/d-galactosamineinduced liver injury in rats (Prevent)	betulinic acid	Antioxidation, hepatoprotection	20 and 50 mg/kg (No dose-effect)	[141]
Lipopolysaccharide/d-galactosamine induced hepatitis in rats (Prevent)	*Tridaxprocumbens*	Antioxidation	300 mg/kg	[142]
Doxorubicin-induced liver injury in rats	*N*-acetylcysteine	Antioxidation, hepatoprotection	10 mg/kg	[143]
Cisplatin-induced liver injury in rats (Prevent)	Tomato juice	Antioxidation, hepatoprotection		[144]
Tert-butyl hydroperoxide-induced liver injury in rats (Prevent)	Propolis	Antioxidation, hepatoprotection	50 and 100 mg/kg (No dose-effect)	[145]
Tamoxifen-induced liver injury in mice (Prevent)	Catechin	Antioxidation	40 mg/kg	[146]
Hepatic steatosis stimulated with tunicamycin (Treatment)	Melatonin	↓ ER stress, expression of miR-23a		[147]
Ethionine-induced liver injury in mice (Prevent)	Melatonin	Antioxidation, hepatoprotection	3 mg/kg	[148]

Many pollutants and toxic substances could cause oxidative stress/damage of liver as mentioned above. Among pollutants and toxins that have been used to model hepatic injury in animals for studying effects of antioxidants/plants on pollutant-induced liver damage, carbon tetrachloride (CCl_4_) is most widely used. In CCl_4_-induced liver injury model, oxidative stress could be provoked, which prompts lipid peroxidation that injure hepatocellular membrane, followed by substantial release of pro-inflammatory chemokines and cytokines, which in consequence of liver damage [10]. A large amount of plants, especially medicinal plants, has been investigated to eliminate the hepatic damage stimulated by CCl_4_. For example, *Coptidis rhizome*, a traditional Chinese medicinal plant used to clear heat and scavenge toxins, belongs to liver meridian in Chinese medicinal practice [149,150]. The effect of *Coptidis rhizome* and its bioactive compound berberine on CCl_4_-induced chronic and acute hepatotoxicity in rats has been thoroughly studied by our research group [10,30,127]. We have found that *Coptidis rhizome* might act as an antioxidant to relieve CCl_4_-induced oxidative stress and hepatic damage. The mechanism may partly be ascribed to the reduced phosphorylation of Erk1/2 expression when exposed to oxidative stress [10]. The effects of some antioxidants/plants on toxic substances-induced liver damage are summarized in Table 5. It is particularly worth noting that Nrf2 could be activated by several antioxidants/plants in dimethylnitrosamine or cadmium induced hepatic injury models [151,152,153]. Antioxidant could induce both modification of inhibitor of Nrf2 (INrf2) cysteine 151 and PKC-mediated phosphorylation of Nrf2 serine 40 to release Nrf2 from INrf2. The dissociated and activated Nrf2 then translocates to the nucleus, binds to ARE and up-regulates antioxidants gene expression, which protects cells and relieves injury induced by oxidative stress [154]. Although most of the studies shown in Table 5 suggested the simultaneous role of these natural products as antioxidative and hepatoprotective agents, the related mechanisms and signal pathways have not yet fully studied.

Accumulating evidence demonstrated that ROS could lead to protein modification, lipid peroxidation, DNA damage and therefore acts as the initiator or promoter of carcinogenesis [155,156,157]. As the first line defense in suppressing tumor initiation, antioxidants are treated as one of the promising strategies to prevent liver cancer. Furthermore, it has been reported that the combination of certain chemotherapeutic drugs and antioxidants could reduce drug resistance, sensitizing the liver cancer cells to chemotherapeutics and therefore improving the efficacy of anti-cancer therapy [158]. Our previous studies demonstrated that *Coptidis rhizome* and berberine are promising agents to fight against liver cancer due to their hepatoprotective and antioxidant properties [155,157,159,160]. In all, cumulative evidence from epidemiological and clinical studies showed that consumption of suitable antioxidants from natural sources may beneficial in fighting against cancer without obvious adverse effects. Besides liver cancer, oxidative injury-associated liver damage induced by other disorders has also been mentioned for confirming the use of antioxidants in the related diseases. For example, it was found that taking catechin from green tea could reduce injury of liver in cholestatic rats induced by bile duct ligation [161]. Allopurinol, a competitive xanthine oxidase inhibitor, has also been used to reduce systemic oxidative stress. The xanthine oxidase over-activity is suggested to play a role in the altered intestinal permeability in cirrhosis, it was found in an open-label pilot study that changes in intestinal permeability correlated to changes in MDA serum values after allopurinol treatment [162]. Additionally, treatment with allopurinol in bile-duct ligation rats and TAA induced liver injury was shown to reduce ROS and thus attenuate brain edema [163]. Effects of certain antioxidants/plants on other substances-induced liver damage are summarized in Table 6, which suggested that some antioxidants possess anti-tumor and hepatoprotective effects collectively *in vivo*, but the relationship and mechanisms need further exploration.

Notably, melatonin, *N*-acethyl-5-metoxytryptamine, a famous hormone synthesized mainly by the pineal gland, has been demonstrated as having striking antioxidant properties in numerous studies. It has the remarkable capability to scavenge both ROS and RNS, and block transcriptional factors of pro-inflammatory cytokines. Recently, it has been applied to the treatment of liver disease in terms of reducing oxidative stress [164]. A variety of liver disease models, such as streptozocin-induced diabetic rats and TAA-induced or bile-duct ligated fibrosis rats, melatonin administration showed hepato-protection partially via improving oxidative damage. As a matter of fact, it has been demonstrated that melatonin is even better antioxidant than vitamin E and C in the contexts of certain disease. A comparative study of the protective effects of melatonin and vitamin E on extra-hepatic bile duct ligation in rats indicated that melatonin is much more efficient than vitamin E in reducing the cholestasis parameters, decreasing lipid peroxidation and restoring anti-oxidative enzymes [165,166]. Further investigations are required to evaluate antioxidant and hepato-protective effect of melatonin in clinic.

**Table 5:** The effects of some antioxidants/plants on toxins-induced liver damage.

Model (Prevent/Treatment)	Antioxidant/Plant	Effects	Dose/(Dose–Effect)	Bioactive Compounds	References
CCl_4_-induced liver damage in rats (Prevent)	*Coptidis rhizome* and berberine	↑ SOD; ↓ ALT, AST, Erk1/2	Berberine: 120 mg/kg b.w. Extract: 800 mg/kg b.w.	Berberine	[10]
CCl_4_-induced liver damage in rats (Prevent)	Friedelin isolated from *Azima tetracantha* leaves	↑ SOD, CAT, GSH, GSH-Px; ↓ ALT, AST, LDH			[59]
CCl_4_-induced liver damage in rats (Treatment)	*N*-butanol fraction of *Actinidias deliciosa* roots	↑ GSH; ↓ ALT, AST, MDA	(Dose–effect)	Oleanolic acid	[167]
CCl_4_-induced liver damage in rats (Prevent)	*Silybum marianum* seeds	↑ GSH; HDL/LDL; hepatoprotection	100 mg/kg b.w.		[168]
CCl_4_-induced liver damage in rats (Prevent)	*Dioclea reflexa* seeds	↑ SOD, CAT; ↓ Transaminases, MDA	5 mg/kg (acute) 2.5 mg/kg b.w. (chronic)		[169]
CCl_4_-induced liver damage in rats (Prevent)	*Morus bombycis*, 2,5-dihydroxy-4,3′-di (β-d-glucopyranosyloxy)-*trans*-stilbene	↓ Lipid peroxidation; hepatoprotection	100, 300 and 500 mg/kg b.w. (No dose–effect)		[170,171]
CCl_4_-induced liver damage in rats (Prevent)	*Nigella sativa*, *Urticadioica*	↑ Antioxidant enzyme; ↓ lipid peroxidation; hepatoprotection	*Nigella sativa*: 0.2 mg/mL *Urtica dioica*: 0.2 mg/mL		[172]
CCl_4_-induced liver damage in rats (Prevent)	*Pleurotusostreatus* (oyster mushroom)	↑ GSH, CAT, SOD, GSH-Px; ↓ ALT, AST, ALP, MDA	200 mg/kg b.w.		[173]
CCl_4_-induced liver damage in rats (Prevent)	*Cytisusscoparius*	↑ GSH, CAT, SOD, GSH-Px, GST, GRD; ↓ ALT, AST, LDH	250 and 500 mg/kg (No dose–effect)		[174]
CCl_4_-induced liver damage in rats (Prevent)	Ethanol extract of *Phellinusmerrillii*	↑ CAT, SOD, GSH-Px; ↓ ALT, AST	0.5, 1 and 2 g/kg b.w. (No dose–effect)		[175]
CCl_4_-induced liver damage in rats (Prevent)	*Ginkgo biloba*	↑ GSH, SOD, CAT, GSH-Px, GRD, albumin; hepatoprotection	25 and 50 mg/kg b.w. (No dose–effect)		[176]
CCl_4_-induced liver damage in mice (Prevent)	Protein isolate from *Phyllanthus niruri*	↑ SOD, CAT; ↓ ALT, ALP; lipid peroxidation	5 mg/kg b.w.		[177]
CCl_4_-induced liver damage in mice (Prevent)	Kahweol and cafestol (Coffee)	↓ ALT, AST, cytochrome P450 2E1, lipid peroxidation	Kahweol or cafestol: 10–100 mg/kg b.w. (Dose–effect)	Kahweol and cafestol	[178]
CCl_4_-induced liver damage in rats (Prevent)	*Cirsium setidens*	↑ GSH-Px; SOD; hepatoprotection	500 mg/kg b.w.		[179]
CCl_4_-induced liver damage in rats (Prevent)	Curcumin and saikosaponin A	↑ SOD, GSH; ↓ MDA; hepatoprotection			[180]
CCl_4_-induced liver damage in rats (Prevent)	Ethanolic extract of *Momordica tuberosa* tubers	Antioxidation, hepatoprotection			[181]
CCl_4_-induced liver damage in rats (Prevent)	Oregano and rosemary	↓ AST, ALT, ALP; antioxidation	20 g/kg b.w.		[182]
CCl_4_-induced liver damage in rats (Prevent)	*Enicostemma axillare*	Antioxidation, hepatoprotection	100 and 200 mg/kg b.w. (No dose–effect)		[139]
CCl_4_-induced liver damage in rats (Prevent)	*Ficuscarica* leaves and fruits, *Morus alba* root barks	↑ CAT, SOD, GSH; ↓ MDA, AST, ALT, ALP	50 and 150 mg/kg b.w. (No dose–effect)		[183]
CCl_4_-induced liver damage in rats (Prevent)	*Podophyllum hexandrum*	↑ GSH, GSH-Px, GRD, SOD, GST; ↓ AST, ALT, LDH	20, 30 and 50 mg/kg b.w. (No dose–effect)		[184]
CCl_4_-induced liver damage in rats (Prevent)	*Ficusreligiosa* roots	↑ CAT, GSH-Px, GRD, SOD, GST; ↓ lipid peroxidation; hepatoprotection			[185]
CCl_4_-induced liver damage in rats (Prevent)	Dehydroabietylamine, *Carthamus tinctorious*	↓ AST, ALT, ALP; antioxidation			[186]
CCl_4_-induced liver damage in rats (Prevent)	Artemetin, *Vitexglabrata*	↑ SOD, CAT, GSH-Px; ↓ AST, ALT, ALP, lipid peroxidation, TB			[187]
CCl_4_-induced liver damage in mice (Prevent)	Blueberry anthocyanins	↑ SOD, CAT, GRD, glycogen; ↓ AST, ALT, MDA			[188]
CCl_4_-induced liver damage in rats (Prevent)	*Matricaria chamomilla*	↑ SOD, CAT, GSH-Px, GSH; ↓ AST, ALT, MDA	50, 100 and 200 mL/kg b.w. (No dose–effect)		[189]
CCl_4_-induced liver damage in mice (Prevent)	*Lysimachia clethroides*	↑ SOD; ↓ AST, ALT, MDA	150, 300 and 600 mg/kg b.w. (No dose–effect)		[190]
CCl_4_-induced liver damage in rats (Prevent)	*Garcinia indica* fruit rind	↑ SOD, CAT, GRD, GSH-Px, GSH; ↓ AST, ALT, MDA	400 and 800 mg/kg b.w. (No dose–effect)		[191]
CCl_4_-induced liver damage in rats (Prevent)	*Agaricus blazei*	↑ GSH, GRD; ↓ AST, ALT, MDA	500 mg/kg b.w.		[192]
CCl_4_-induced liver damage in rats (Prevent)	*Nerium oleander* flowers	↑ SOD; ↓ AST, ALT, ALP, MDA	100, 200 and 400 mg/kg b.w. (No dose–effect)		[193]
CCl_4_-induced liver damage in rats (Prevent)	*Hybanthus enneaspermus*	↓ AST, ALT, ALP, TB; antioxidation	200 and 400 mg/kg b.w. (No dose–effect)		[194]
CCl_4_-induced liver damage in mice (Treatment)	Anthocyanins in black rice bran	↑ SOD, GSH-Px; hepatoprotection	200, 400 and 800 mg/kg b.w. (No dose–effect)		[195]
CCl_4_-induced liver damage in rats (Prevent)	*Roureainduta*	↑ SOD, CAT, GSH, GSH-Px; ↓ AST, ALT, total bilirubin;	500 mg/kg b.w.		[196]
CCl_4_-induced liver damage in rats (Prevent)	Proanthocyanidins extracted from grape seeds	↑ SOD, GSH, GSH-Px, CAT; ↓ lipid accumulation, liver injury, DNA damage	400 mg/kg b.w.	Proanthocyanidins	[197]
CCl_4_-induced liver damage in mice (Prevent)	*Veronica ciliata*	↑ SOD, GSH; ↓ ALT, AST, ALP	150, 300 and 600 mg/kg b.w. (No dose–effect)		[198]
CCl_4_-induced liver damage in rats (Prevent)	*Subereamollis*	↑ SOD, GSH, GSH-Px, CAT; ↓ ALT, AST, ALP, MDA	100, 200 and 400 mg/kg b.w. (No dose–effect)		[199]
CCl_4_-induced liver damage in rats (Prevent)	*Solanum xanthocarpum* leaves	↑ SOD, CAT, GSH, GST; ↓ ALT, AST, ALP, LDH	100 and 200 mg/kg b.w. (No dose–effect)		[200]
CCl_4_-induced liver damage in rats (Prevent)	*Allopurinol*	Modulation of NF-κB, cytokine production and oxidative stress	50 mg/kg b.w.		
CCl_4_ and H_2_O_2_ induced liver damage in goat (Prevent)	*Ocimumbasilicum*, *Trigonellafoenum-graecum*	Antioxidation			[201]
TAA-induced liver injury (Prevent)	Genistein	↑ GSH; ↓ MDA, ALT, AST, TB	0.5, 1.0 and 2.0 mg/kg b.w. (No dose–effect)		[202]
TAA-Induced liver Cirrhosis in rats (Prevent)	*Andrographis paniculata* Leaf	Hepato-protection, ↓ ROS, LDH	250 and 500 mg/kg b.w. (No dose–effect)		[203]
TAA-induced hepatotoxicity in rats (Prevent)	coriander	Antioxidant; ↓ ALT, AST, ALP, TBARS, MPO, NO		Phenolic compounds	[204]
TAA-induced fibrosis in mice (Treatment)	*Ger-Gen-Chyn-Lian-Tang*	Antioxidant; anti-fibrosis; modulation on TGF-β/TGF-β receptor signaling	100 and 300 mg/kg b.w. (Dose–effect)		[205]
TAA-induced hepatotoxicity in rats (Treatment)	*Trigonella foenum*-*graecum*	Antioxidant; hepato-protection; ↓ ALP, MDA			[206]
TAA-induced hepatotoxicity in rats (Treatment)	Allopurinol	Regulating cellular redox-sensitive transcription factors			[163]
Cigarette smoke-induced oxidative damage in liver of rats (Treatment)	*Sesbania grandiflora* leaves	↑ SOD, GSH, GSH-Px, CAT, GST, GRD, glucose-6-phosphate dehydrogenase; ↓ AST, ALT, ALP	1000 mg/kg b.w.		[207]
Cigarette smoking induced oxidative damage in liver of mice (Prevent)	Vitamin E and selenium	↑ GSH-Px, Se-GSH-Px			[208]
Atrazine exposure rats (Prevent)	Vitamin E	↑ SOD, GSH-Px, CAT, GST; ↓ lipid peroxidation			[209]
Methidathion-induced liver injury in rats (Prevent)	Vitamins C and E	↓ AST, ALT, ALP, MDA;	Vitamin E: 50 mg/kg b.w.;Vitamin C: 20 mg/kg b.w.		[210]
Pesticide (chlorpyriphos and cypermethrin) induced hepatic damage in mice (Prevent)	Black tea	↑ SOD, GSH, GSH-Px, CAT, GRD, GST; ↓ AST, ALT, ALP	200 mg/mL b.w.		[211]
Polychlorinated biphenyls induced hepatic damage in rats (Prevent)	α-Tocopherol	Antioxidation	50 mg/kg. b.w.		[212]
Aflatoxin-induced hepatic injury in rats (Prevent)	*Urticadioica* seed	↑ SOD, GSH-Px, CAT, GRD, GST; ↓ lipid peroxides, hydroxyl radical and hydrogen peroxides	2 mL/rat/day		[213]
Thioacetamide-induced hepatic damage in rats (Prevent)	eugenol	↑ COX-2; ↓ AST, ALT, ALP, bilirubin, CYP2E1, lipid peroxidation; antioxidation	10.7 mg/kg b.w.		[214]
Lead-induced liver damage in rats (Prevent)	Ginger	↑ SOD, CAT; ↓ MDA	100 mg/kg b.w.		[215]
Dimethylnitrosamine-induced hepatic damage in rats (Prevent)	Anthocyanins from purple sweet potato	↑ Nrf2, NADPH, GSH, GST; ↓ yclooxygenase-2, MDA	50, 100 and 200 mg/kg b.w. (No dose–effect)	Anthocyanins	[151]
Cadmium-induced hepatic injury in rats (Prevent)	Heated garlic juice, ascorbic acid	↑ Nrf2, SOD, CAT; ↓ MDA	Heated garlic juice: 100 mg/kg b.w.; Ascorbic acid: 100 mg/kg b.w.	Ascorbic acid	[152]
Potassium bromate-induced hepatotoxicity of rat (Prevent)	*Launaea procumbens*	↑ SOD, CAT, GSH, GSH-Px, GRD, GST	200 mg/kg b.w.		[216]
Dimethylnitrosamine induced liver fibrosis in rats (Prevent)	*Platycodi radix* root	↑ Nrf2, heme oxygenase-1, NADPH, NQO1, GST; ↓ ALT, AST; anti-fibrotic action	200 mg/kg b.w.	Changkil	[153]
As_2_O_3_-induced hepatotoxicity in cat (Prevent)	Resveratrol	↑ GSH; ↓ ROS, MDA	3 mL/kg b.w.		[217]
Sodiumarsenite induced liver damage in rats (Prevent)	*Emblica officinalis*	Antioxidation	500 mg in 0.1 mL water, 100 g b.w.		[218]
Trichloroacetic acid induced liver injury in rats (Prevent)	Date palm fruit	↑ SOD, CAT, GSH-Px; ↓ MDA	0.5 and 2 g/L b.w. (No dose–effect)		[219]

**Table 6:** Effects of some antioxidants/plants on other related liver disease.

Stress (Prevent/Treatment)	Antioxidant/Plants	Effects	Dose (Dose–Effect)	Bioactive Compounds	References
Human liver cancer cell line	*Morinda pubescens* leaves	Antioxidation, cytotoxicity	25, 50, 100 and 250 μg/mL b.w. (Dose–effect)	Hyoscyamine	[220]
Liver cancer of rats (Prevent)	*Chlorella vulgaris*	Antioxidation, antitumour	50, 150 and 300 mg/mL b.w. (Dose–effect)		[221]
Hepatocellular carcinoma	*Caesalpinia bonducella* leaves	↑ SOD, GSH, CAT; ↓ MDA, AST, ALT, ALP; anticancer		Flavonoids, triterpenoids	[222]
Liver cancer of mice (Prevent)	*Pleurotus pulmonarius* (edible mushroom)	Antioxidation, anti-tumor			[158]
Rat with secondary biliary cirrhosis (Prevent)	Silybin	Antioxidation	0.4 g/kg b.w.		[223]
Cholestatic rats with bile duct ligation (Treatment)	Green tea catechin	Antioxidation, reducing hepatic fibrosis	50 mg/kg b.w.		[161]
Bile duct-ligated cholestatic rats (Treatment)	Epigallocatechin-3-gallate	Anti-fibrotic effects, ↓ phosphorylation of Smad2/3 and Akt	5 mg/kg b.w.		[224]
Bile duct-ligated cholestatic rats (Treatment)	*Holothuria arenicola*	↑ SOD, GSH, GST, CAT; ↓ MDA, AST, ALT, ALP	200 mg/kg b.w.	Phenolic compounds, chlorogenic acid, pyrogallol, rutin, coumaric acid	[225]
Bile-duct ligated Rats (Treatment)	Garlic	↑ GSH; ↓ LDH, TB, MDA, MPO; ↓ TNF-α, TGF-β, MMP-13			[226]
Bile-duct ligated Rats (Treatment)	thymoquinone	↑ SOD, GSH-Px; ↓ MDA	50 mg/kg b.w.		[227]
Bile-duct ligated Rats (Treatment)	*N*-acetylcysteine	↑ GSH, CAT; ↓ MDA, ALT	300 mg/kg b.w.		[228]
Bile-duct ligated Rats (Prevent)	*Phaseolus trilobus*	↑ SOD; ↓ AST, ALT, ALP, LDH, TB, TBARS;	125, 250 and 500 mg/kg b.w. (Dose–effect)		[229]
Bile-duct ligated Rats (Treatment)	Melatonin	↓ TBARS, MPO	10 and 100 mg/kg b.w. (Dose–effect)		[230]
Ischemia/reperfusion in obese rats with fatty liver	Melatonin	↑ Antioxidant enzymes; ↓ AST, ALT, MAD, NOx metabolites	10 mg/kg b.w.		[231]
Bile-duct ligated Rats (Treatment)	*Allopurinol*	↓ ROS, brain edema	100 mg/kg b.w.		[232]
Restraint stress-induced liver injury in mice (Prevent)	*Astragali radix* and *Salviae radix*	Antioxidation, hepatoprotection	50, 100 and 200 mg/kg b.w. (No dose–effect)	Myelophil	[233]

## 4. Current Anti-Oxidative Therapy in Clinical Trials

Clinical trials are extremely vital and indispensable for the development of anti-oxidative therapy. We looked up the related information of current anti-oxidative therapy in clinic at http://www.ClinicalTrials.gov website. Vitamins, especially vitamin E, are the most frequently studied antioxidant as dietary supplement in clinical trials for liver disease, primarily in phase 2/3. Some other nutritional antioxidants such as zinc and Coenzyme Q10 were studied in phase 2. Compounds including silymarin, metadoxine, *N*-acetylcystein, propofol, and mitoquinone mesylate, which partially act as antioxidant, have been used as drugs or supplement for liver disease. Some of them, such as silymarin, metadoxine and *N*-acetylcysteine, are studied for NAFLD or NASH or ALD in phase 4. For example, the application of antioxidants supplement consisted of siliphos, selenium, methionine, and alpha lipoic acid has been approved in patients with fatty liver and non-alcoholic steatohepatitis in Mexico. Plants and foods such as ginger, green tea extract, and chocolate have been adopted as food supplement for their anti-oxidative properties for liver disease. Furthermore, quercetin and resveratrol, two well-known bioactive compounds isolated from plants, have been studied as food supplement as antioxidants for liver disease in phase 3. Despite certain promising results have obtained in clinical trials, anti-oxidative therapy still has a long way to go. As a matter of fact, many antioxidants are highly effective for prevention or treatment in animal models, but in humans it does not appear to be effective for the treatment of established disease. For example, anti-TNF, which shows desirable treatment effects in animal model, appears not to be effective in patients with acute alcoholic hepatitis. Therefore, translational research is highly important for the application of antioxidant therapy in clinic. In the future, natural plants and bio-active compounds isolated from plants as well as endogenous antioxidants such as melatonin, which have shown strong anti-oxidative ability and hepato-protection effects, should be studied by clinic trials with large patient samples and longer duration time.

## 5. Conclusions and Prospects

Anti-oxidative therapy, mainly using natural and synthetic antioxidants, represents a reasonable therapeutic approach for the prevention and treatment of liver diseases due to the role of oxidative stress in contributing to initiation and progression of hepatic damage. However, although concept of anti-oxidative therapy has been raised for decades and intensive efforts have been paid, there is a long way to go for the application of antioxidants in liver disease. In current clinical trials, mechanisms by which drugs or compounds treat liver disease might partly attribute to anti-oxidative ability, but plain antioxidants mainly used as dietary supplement to prevent the progress of disease or improve the outcome of patients might also be effective. The complex role of oxidative stress in physiological and pathological processes, lacking studies of underlying mechanisms in humans, and other difficulties in translational research are challenges ahead. In current studies, intervention of antioxidants is explored widely in prevention models rather than treatment model, without elaborated underlying mechanism investigation. For natural plants study, the dose used, especially content of antioxidants, is always blurry, not to mention the shift dose for humans. For those studies in which dose–effect has been investigated, only small portion of plants antioxidant showed dose–effect manner for reducing liver injury, suggesting the complex role of oxidative stress in pathogenesis. In animal study, antioxidants are given to animals via oral or intraperitoneal injection. The route of administration is also an influence for absorption and bio-availability of antioxidants. Additionally, since liver is a central organ for metabolism, oxidative stress in liver diseases interacts with many other diseases such as kidney failure and diabetes, certain models in animal study should be improved. These limitations in current study might result in antioxidants that showed desirable effects for prevention or treatment in animal models, but in humans they do not appear to be effective for the treatment of established disease, which is a barrier for the development of anti-oxidative therapy in clinic. Therefore, translational research is of great importance for anti-oxidative therapy. Considering ROS and oxidative stress act positively in certain circumstances and the difference between animals and humans, the effective dose and safe dose, duration of treatment, absorption and bio-availability of antioxidants require thorough investigation. Furthermore, in the future, large-scale samples and appropriate duration of anti-oxidative treatment for liver diseases should be performed. 

## Abbreviations

AC: Autoimmune cholestatic liver diseases
ADH: Alcohol dehydrogenase
AIH: Autoimmune hepatitis
ALD: Alcoholic liver disease
ALP: Alkaline phosphatase
ALT: Alanine transaminase
ALDH: Aldehyde dehydrogenases
ARE: Antioxidant response element
AST: Aspartate aminotransferase
BHA: Butylated hydroxyanisole
bw: Body weight
CAT: Catalase
CCl_4_: Carbon tetrachloride
ER: Endoplasmic reticulum
GSH-Px: Glutathione peroxidase
GSH: Glutathione
GRD: Glutathione reductase
GST: Glutathione S-transferase
HDL: High density lipoprotein
HCV: Hepatitis C virus
IL-6: Interleukin 6
INH: Anti-tuberculosis agent isoniazid
iNOS: Inducible nitric oxide synthase (iNOS)
INrf2: Inhibitor of Nrf2
IKKβ: IκB kinase-β
IRS: Insulin receptor substrate
JNK: c-Jun N-terminal kinases
Keap1: kelch-like ECH-associated protein-1
LDH: lactate dehydrogenase
LDL: Low density lipoprotein
MDA: Malondialdehyde
MEOS: Microsomal ethanol oxidizing system
NADPH: Nicotinamide adenine dinucleotide phosphate-oxidase
NAFLD: Non-alcoholic fatty liver disease NAFLD
NO: Nitric Oxide
NQO1: NAD(P)H Dehydrogenase, Quinone 1
Nrf1: Nuclear respiratory factor 1
Nrf2: Erythroid 2-related factor 2
PKC: protein kinase C
PPARα: Peroxisome proliferator activated receptor α
RNS: Reactive nitrogen species
ROS: Reactive oxygen species (ROS)
SOD: Superoxide dismutases
TAA: Thioacetamide
TB: Total bilirubin
TBARS: Thiobarbituric acid-reactive substances
TNF: Tumor necrosis factor
